# Use of Oral Corticosteroids and Risk of Hip Fracture in the Elderly in a Case-Control Study

**DOI:** 10.3389/fphar.2017.00625

**Published:** 2017-09-11

**Authors:** Shih-Wei Lai, Cheng-Li Lin, Kuan-Fu Liao

**Affiliations:** ^1^College of Medicine, China Medical University Taichung, Taiwan; ^2^Department of Family Medicine, China Medical University Hospital Taichung, Taiwan; ^3^Management Office for Health Data, China Medical University Hospital Taichung, Taiwan; ^4^Graduate Institute of Integrated Medicine, China Medical University Taichung, Taiwan; ^5^College of Medicine, Tzu Chi University Hualien, Taiwan; ^6^Department of Internal Medicine, Taichung Tzu Chi General Hospital Taichung, Taiwan

**Keywords:** elderly, hip fracture, oral corticosteroids

## Abstract

**Aim:** Little is known regarding the relationship between use of oral corticosteroids and hip fracture in the elderly in Taiwan. The aim of the study was to examine this issue.

**Methods:** A retrospective population-based case-control study using the database of the Taiwan National Health Insurance Program (2000–2013) was conducted. We identified 4538 individuals aged ≥ 65 years with newly diagnosed hip fracture as the cases. We randomly selected 4538 individuals without hip fracture as the control subjects. The cases and the control subjects were matched with sex, age, comorbidities, and the year of index date. Individuals who never had a prescription for oral corticosteroids were defined as never use. Individuals who ever had at least one prescription for oral corticosteroids were defined as ever use. The odds ratio (OR) and 95% confidence interval (CI) of hip fracture associated with oral corticosteroids use was estimated by a multivariable unconditional logistic regression analysis.

**Results:** After adjustments for potential confounding factors, the multivariable logistic regression model showed that the adjusted OR of hip fracture was 1.17 for individuals with ever use of oral corticosteroids (95%CI 1.08, 1.28), compared to those with never use of oral corticosteroids. An sub-analysis showed that for every 1-mg increase in cumulative dose of oral corticosteroids, the adjusted OR of hip fracture was 1.01 (95% CI 1.01, 1.02). The adjusted ORs were 1.31 (95% CI 1.17, 1.47) for cumulative exposure to oral corticosteroids ≥ 3 months and 1.09 (95% CI 0.98, 1.20) for cumulative exposure < 3 months.

**Conclusion:** We conclude that oral corticosteroids use is associated with a trivial but statistically significant increase in risk of hip fracture in Taiwan. Additionally, the results suggest that there are dose-response and duration-response effects of oral corticosteroids on the risk of hip fracture. The results confirm our understanding of oral corticosteroid-associated hip fracture in the elderly.

## Introduction

Hip fracture persists to be an important public health problem in the elderly worldwide due to its large socioeconomic burden, substantial morbidity and mortality. A systematic Review by Mohd-Tahir and Li (2017) reported that healthcare costs associated with hip fracture accounted for more than one third of gross domestic product (GDP) in some counties of Asia. A observational study in Taiwan by Huang et al. (2016) reported that if the hip-fractured older people had more comorbidities, they would be more likely to have poor outcomes on psychological conditions, such as cognitive impairment and depression. A prospective cohort study in Taiwan by Hung et al. (2014) reported that the hip-fractured older people were at higher risk of mortality compared with the non-fractured matched older people.

It is well-known that individuals with corticosteroids use are at an excess risk of osteoporotic fractures. A population-based case-control study in Denmark by Vestergaard et al. (2003) reported that corticosteroid users were at increased risk of hip fracture compared to never users. A meta-analysis by Kanis et al. (2004) reported that corticosteroids use correlated with high risk of hip fracture in the elderly aged 65–85 (relative risk 2.13–2.98).

Due to the following points: (a) hip fracture remains to be a public health problem in the elderly, (b) the hospitalization number of hip fracture and the total costs of hip fracture hospitalization substantially increased among the elderly in Taiwan reported by Chan et al. (2013) (c) to date, data on the effect of corticosteroids use and the risk of hip fracture is limited in the elderly in Taiwan, we made a rational link between corticosteroids use and hip fracture in the elderly. Therefore, in the present study we used the claim data from the Taiwan National Health Insurance Program to test the following objectives: (a) the relationship between use of oral corticosteroids and hip fracture in the elderly; (b) the dose-response and duration-response effects of oral corticosteroids on the risk of hip fracture.

## Materials and Methods

### Data Source and Study Design

Taiwan is an independent country with more than 23 million residents (Chao et al., 2015; Chen et al., 2015; Ho and Chang, 2015; Hsiao et al., 2015; Hung and Ku, 2015; Chen and Wu, 2016; Chen S.Y. et al., 2016; Chen Y.F. et al., 2016; Hsieh et al., 2016; Hsu and Yin, 2016; Huang and Chang, 2016; Lin and Lin, 2016; Maa and Leu, 2016; Ooi, 2016; Yu et al., 2016). The Taiwan National Health Insurance Program began in March 1995, and now it has covered around 99% of the residents living in Taiwan (National Health Insurance Research Database, 2017). The details of the program were written down in previous studies (Lai et al., 2010; Kuo et al., 2015; Yang et al., 2015; Chen H.Y. et al., 2016; Tsai et al., 2016). A retrospective population-based case-control study using the database of the Taiwan National Health Insurance Program (2000–2013) was conducted. The study was approved by the Research Ethics Committee of China Medical University and Hospital in Taiwan (CMUH-104-REC2-115).

### Identification of Cases and Control Subjects

**FIGURE 1:**
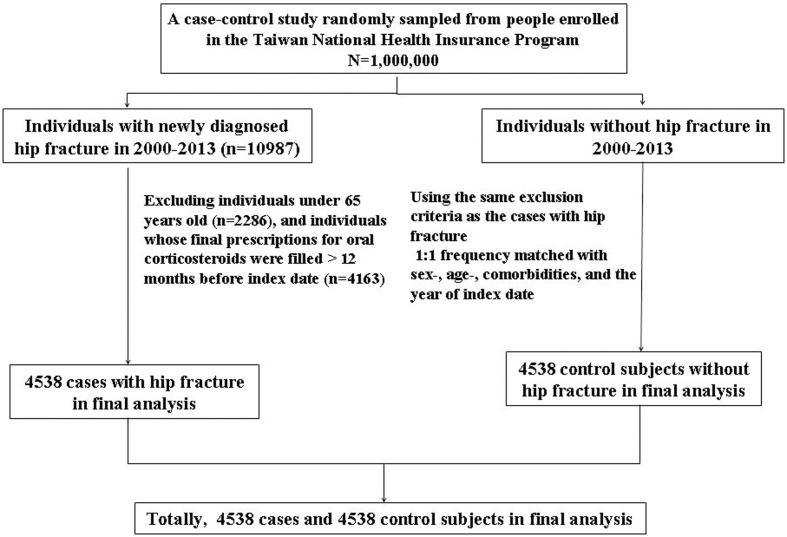
Flow chart shows selection process of cases with hip fracture and control subjects.

We identified individuals aged ≥ 65 years with newly diagnosed hip fracture in 2000–2013 as the cases with hip fracture [the International Classification of Diseases (ICD) 9th Revision, ICD-9 code 820]. The index date was defined as the date of cases being diagnosed with hip fracture. We assigned a random number for each individual without a diagnosis of hip fracture in the database by generating random numbers between 0 and 1 that were distributed uniformly. Individuals without hip fracture randomly selected were assigned as the control subjects who were matched in terms of sex, age (5-year interval), and comorbidities. The enrollment date for the control subjects was matched with the same year of cases with hip fracture, while the month and day were randomly assigned. The sample size of the control subjects was onefold as the cases with hip fracture. Therefore, it is unlikely that the control subjects were chosen intentionally (**Figure 1**).

### Confounding Factors

Comorbidities potentially associated with hip fracture before the index date were included as follows: alcohol-related disease, cardiovascular disease, chronic kidney disease, chronic obstructive pulmonary disease, diabetes mellitus, hyperlipidemia, and hypertension. All comorbidities were used based on ICD-9 codes. To increase the analysis accuracy, individuals who needed to have the same diagnosis for at least three consecutive records in the ambulatory care visit and/or at least one time of hospitalization diagnosis could be included in the study. Principal diagnosis and secondary diagnosis were used equally. Therefore, hip fracture and other comorbidities were addressed for three or more records in the ambulatory care and/or at least one record during hospitalization. Such strict criteria have been used in the previous study (Lai et al., 2015d, 2017a,b,c).

### Measurements of Corticosteroids Use

The measurements of corticosteroids use were adapted from previous studies (Lai et al., 2015a, 2016, 2017c). Shortly speaking, there is much difficulty in measuring the dosage of topical corticosteroids use or inhaled corticosteroids use. Individuals with long-time use of injected corticosteroids at outpatient department are also rarely found. To measure the dosage accurately, only oral corticosteroids were included for statistical analysis. Therefore, topical, inhaled, and injected corticosteroids were combined together as other forms of corticosteroids for adjustments. The availability of oral corticosteroids in Taiwan was listed as follows: cortisone, dexamethasone, fludrocortisone, methylprednisolone, prednisolone, and triamcinolone. We colleted the prescription histories of oral corticosteroids before the index date. If individuals’ final prescriptions for oral corticosteroids were filled > 12 months before the index date, they were excluded from the study. Therefore, only individuals whose final prescriptions for oral corticosteroids were filled within 12 months before the index date could be included for statistical analysis. Individuals who never had a prescription for oral corticosteroids were defined as never use. Individuals who had at least one prescription for oral corticosteroids were defined as ever use.

### Statistical Analysis

The Chi-square test was used to compare the differences of sex, age, oral corticosteroids use, other forms of corticosteroids use, and comorbidities between the cases and the control subjects. The *t*-test was used to compare the differences of mean ages and mean days of exposure to oral corticosteroids between the cases and the control subjects. The univariable and multivariable unconditional logistic regression models were used for statistical analysis. Variables which were found to be statistically significant in a univariable model were further examined in a multivariable model to measure the odds ratio (OR) and 95% confidence interval (CI) of hip fracture associated with oral corticosteroids use. We conducted the analysis to examine the dose-response and duration-response effects of oral corticosteroids on the risk of hip fracture. We conducted an additional analysis to examine the interaction effects between oral corticosteroids use and polypharmacy (use of five or more drugs) on the risk of hip fracture. All data processing and statistical analyses were performed with the SAS software version 9.2 (SAS Institute, Inc., Cary, NC, United States). A two-tailed *P*-value < 0.05 was considered statistically significant.

## Results

### Descriptive Information of the Study Population

**Table 1** shows 4538 new cases with hip fracture and 4538 control subjects without hip fracture (**Table 1**). The cases and the control subjects had similar distribution of sex. Females constituted a higher proportion in cases and control subjects (58%). The mean ages (standard deviation) were 79.7 (7.2) years in cases and 79.3 (7.1) years in control subjects, with statistical significance (*t*-test, *P* = 0.01). The mean durations (standard deviation) of exposure to oral corticosteroids were 263.6 (606.2) days for the cases and 232 (555.5) days for the control subjects, without statistic significance (*t*-test, *P* = 0.10). The cases were more likely to have a higher proportion of ever use of oral corticosteroids than the control subjects (42.1% vs. 38.5%, Chi-square test, *P* < 0.001). There were no significant differences of ever use of other forms of corticosteroids and comorbidities between the cases and the control subjects (Chi-square test, *P* > 0.05).

**Table 1:** Descriptive information of cases with hip fracture and control subjects in the elderly.

	Hip fracture				
		
	No *N* = 4538		Yes *N* = 4538		
			
Variable	*n*	(%)	*n*	(%)	*P*-value^∗^
Sex					0.88
Female	2651	(58.4)	2658	(58.6)	
Male	1887	(41.6)	1880	(41.4)	
Age group (years)					0.84
65–74	1275	(28.1)	1251	(27.5)	
75–84	2166	(47.7)	2176	(48.0)	
≥85	1097	(24.2)	1111	(24.5)	
Age (years), mean (standard deviation)^†^	79.3	(7.1)	79.7	(7.2)	0.01
Days of exposure to oral corticosteroids, mean (standard deviation)^†^	232	(555.5)	263.6	(606.2)	0.10
Ever use of oral corticosteroids	1745	(38.5)	1909	(42.1)	<0.001
Ever use of other forms of corticosteroids	1974	(43.5)	1995	(44.0)	0.66
Comorbidities before index date					
Alcohol-related disease	81	(1.78)	68	(1.50)	0.28
Cardiovascular disease	2984	(65.8)	2949	(65.0)	0.44
Chronic kidney disease	385	(8.48)	371	(8.18)	0.59
Chronic obstructive pulmonary disease	1512	(33.3)	1521	(33.5)	0.84
Diabetes mellitus	992	(21.9)	1046	(23.1)	0.17
Hyperlipidemia	1319	(29.1)	1317	(29.0)	0.96
Hypertension	3483	(76.8)	3459	(76.2)	0.55


*Data are presented as the number of subjects in each group with percentages given in parentheses, or mean with standard deviation given in parentheses. ^∗^Chi-square test, and ^†^*t*-test comparing subjects with and without hip fracture.*

### Association of Hip Fracture with Oral Corticosteroids Use, Other Forms of Corticosteroids Use, and Comorbidities in the Elderly

After adjustments for potential confounding factors, the multivariable logistic regression model showed that the adjusted OR of hip fracture was 1.17 for individuals with ever use of oral corticosteroids (95% CI 1.08, 1.28), compared to individuals with never use of oral corticosteroids (**Table 2**). In addition, for every 1-year increase in age, the adjusted OR of hip fracture was 1.01 (95% CI 1.00, 1.01).

**Table 2:** Odds ratio and 95% confidence interval of hip fracture associated with corticosteroids use, other forms of corticosteroids, and comorbidities in the elderly.

	Crude^†^		Adjusted^†^	
		
Variable	Odds ratio	(95% CI)	Odds ratio	(95% CI)
Sex (male vs. female)	0.99	(0.91, 1.08)		
Age (per 1 year)	1.01	(1.00, 1.01)	1.01	(1.00, 1.01)
Oral corticosteroids (never use as a reference)				
Ever use	1.16	(1.07, 1.26)	1.17	(1.08, 1.28)
Other forms of corticosteroids (never use as a reference)				
Ever use	1.02	(0.94, 1.11)		
Comorbidities before index date (yes vs. no)				
Alcohol-related disease	0.84	(0.61, 1.16)		
Cardiovascular disease	0.97	(0.89, 1.05)		
Chronic kidney disease	0.96	(0.83, 1.12)		
Chronic obstructive pulmonary disease	1.01	(0.93, 1.10)		
Diabetes mellitus	1.07	(0.97, 1.18)		
Hyperlipidemia	1.00	(0.91, 1.09)		
Hypertension	0.97	(0.88, 1.07)		


*^†^Variables found to be statistically significant in a univariable model were further examined in a multivariable model. Only age and oral corticosteroids could be included for adjustments.*

### Association of Hip Fracture with Cumulative Dosage of Oral Corticosteroids Use in the Elderly

We conducted an analysis for the dose-response effect of oral corticosteroids on the risk of hip fracture (**Table 3**). After adjustments for potential confounding factors, for every 1-mg increase in cumulative dose of oral corticosteroids, the adjusted OR of hip fracture was 1.01 (95% CI 1.01, 1.02), compared to individuals with never use of oral corticosteroids.

**Table 3:** Association of hip fracture with cumulative dosage of oral corticosteroids use in the elderly.

Variable	Case number/control number	Crude OR	(95% CI)	Adjusted OR^†^	(95% CI)
Never use of oral corticosteroids as a reference	2629/2793	1.00	(Reference)	1.00	(Reference)
Cumulative dosage of oral corticosteroids use (increase in dosage for per mg)	1909/1745	1.01	(1.01, 1.02)	1.01	(1.01, 1.02)


*^†^Variables found to be statistically significant in a univariable model were further analyzed in a multivariable model. Adjusted for age.*

### Association of Hip Fracture with Cumulative Duration of Oral Corticosteroids Use in the Elderly

We conducted an analysis for the duration-response effect of oral corticosteroids on the risk of hip fracture (**Table 4**). When stratified by cumulative duration of usage, the adjusted OR of hip fracture was 1.09 (95% CI 0.98, 1.20) when cumulative exposure to oral corticosteroids < 3 months. The adjusted OR increased to 1.31 (95% CI 1.17, 1.47) when cumulative exposure to oral corticosteroids ≥ 3 months, compared to individuals with never use of oral corticosteroids.

**Table 4:** Association of hip fracture with cumulative duration of oral corticosteroids use in the elderly.

Variable	Case number/control number	Crude OR	(95% CI)	Adjusted OR^†^	(95% CI)
Never use of oral corticosteroids as a reference	2629/2793	1.00	(Reference)	1.00	(Reference)
Cumulative duration of oral corticosteroids use					
<3 months	1081/1069	1.07	(0.97, 1.19)	1.09	(0.98, 1.20)
≥3 months	828/676	1.30	(1.16, 1.46)	1.31	(1.17, 1.47)


*^†^Variables found to be statistically significant in a univariable model were further analyzed in a multivariable model. Adjusted for age.*

We made a further analysis to show the interaction effects between oral corticosteroids use and polypharmacy on the risk of hip fracture (Table not shown). When compared to individuals with never use of oral corticosteroids and without polypharmacy, the adjusted OR of hip fracture was 1.15 (95% CI 1.03, 1.27) among individuals with ever use of oral corticosteroids alone and without polypharmacy. The adjusted OR increased to 1.36 (95% CI 1.21, 1.54) among individuals with ever use of oral corticosteroids and polypharmacy.

## Discussion

In the present study, we observed that oral corticosteroid use correlated with a trivial but statistically significant increase in odds of hip fracture, compared to never use (adjusted OR 1.17). To reduce the confounding effects caused by comorbidities, the cases and the control subjects were matched with comorbidities. There were no significant differences of ever use of other forms of corticosteroids and comorbidities between the cases and control subjects (**Table 1**). Therefore, the increased odds of hip fracture could not be totally attributed to the confounding effects caused by other forms of corticosteroids and comorbidities.

In a further analysis, we observed that the odds of hip fracture increased with the cumulative dosage of oral corticosteroid use (**Table 3**), which was compatible with previous studies showing that increasing cumulative dosage of corticosteroid use was associated with increased odds of hip fracture (Van Staa et al., 2000; Vestergaard et al., 2003). These findings suggest that there seems to be a dose-response relationship between oral corticosteroid use and the risk of hip fracture. That is, the higher the cumulative dosage of oral corticosteroid, the greater risk of hip fracture.

In a further analysis, we observed that despite not reaching statistic significance, the risk of hip fracture became slightly higher when cumulative exposure to oral corticosteroids < 3 months (adjusted OR 1.09). This finding was partially compatible with Van Staa et al. (2002) meta-analysis showing that the fracture risk increased more markedly within 3–6 months of initiating oral corticosteroid use. These findings suggest that the risk of hip fracture might increase within the first months of initiating oral corticosteroid use.

In addition, we observed that the odds of hip fracture significantly increased to 1.31 when cumulative exposure to oral corticosteroids ≥ 3 months (**Table 4**), which was compatible with previous studies showing that there was a duration-response relationship between oral corticosteroid use and the risk of hip fracture (Van Staa et al., 2000; Steinbuch et al., 2004). That is, the longer of exposure to oral corticosteroid, the greater risk of hip fracture. A cross-sectional study by Baron et al. (1999) showed that bone mineral density was lower among post-menopausal women using long-term corticosteroids. A cross-sectional study by Shiga et al. (2009) showed that increased bone resorption and decreased bone formation of hip contributed to a greater risk of hip fracture for older women with osteoporosis. Based on the above review and discussion, we confirm previous observational studies that oral corticosteroid-induced osteoporosis might be associated with a trivial but statistically significant increase in the risk of hip fracture in the elderly.

In a previous study, we observed that polypharmacy (use five or more drugs) was associated with increased risk of hip fracture in the elderly (Lai et al., 2010). In a further analysis, we observed that individuals with ever use of oral corticosteroids alone and without polypharmacy remained to have a trivial but statistically significant increase in odds of hip fracture (adjusted OR 1.15). This finding suggests that oral corticosteroids use has an additional effect on the risk of hip fracture, independent of polypharmacy. If individuals concurrently had oral corticosteroids use and polypharmacy, the odds would markedly increase to 1.36. This finding suggests that there is an interaction effect between oral corticosteroids use and polypharmacy on the risk of hip fracture in the elderly.

Some limitations should be discussed in the present study. First, due to the inherent limitation of claim data, there was no record on bone mineral density. We could not examine the status of bone mineral density at the time of hip fracture. That is why we were unable to include osteoporosis for statistical analysis. However, it indicates a research direction on the status of bone mineral density when the elderly developed hip fracture. Second, due to the same limitation, we were unable to make sure whether patients really used oral corticosteroids or not. Therefore, the prescription histories of oral corticosteroids were used for instead. Third, due to the same limitation, there was no record on the indications why oral corticosteroids were prescribed. Thus, we could not explain the high rates of ever use of oral corticosteroids both in cases with hip fracture and in control subjects (42.1% vs. 38.5%). Fourth, we should examine the interaction between oral corticosteroids and other bone-related medications, or the interaction between oral corticosteroids and other falling-related medications. Furthermore, there is much difficulty in designing such a case-control study which needs to include all concomitant medications for adjustments. The more appropriate option was to examine the relative risk of hip fracture associated with one potential drug. That is why we only included oral corticosteroids for statistical analysis. However, it indicates a research direction on the relationship between all concomitant medications in detail and the risk of hip fracture. Fifth, due to unable to obtain specific ICD-9 codes for falls, gait and balance disorders, we were unable to include these disorders for statistical analysis. This point has been addressed in a previous study (Lai et al., 2015b). Sixth, although the diagnosis of all comorbidities was based on ICD-9 codes, the accuracy of ICD-9 codes has been fully discussed in previous studies (Lin et al., 2013; Lai et al., 2015b,c; Shen et al., 2016; Liao et al., 2017a,b; Hung et al., 2017).

Despite the above limitations, some strengths of the present study deserve discussion. Although it is well-known that corticosteroids use creates increased risk of osteoporotic fractures, this is a straight-forward study to examine the relationship between use of oral corticosteroids and hip fracture in the elderly in Taiwan. We present confirmatory data from Taiwan on this adverse drug reaction. We emphasize the dose-response and the duration-response effects of oral corticosteroids on the risk of hip fracture. The topic is an important one and deserves a practical value. The used methodology (case-control study) and the analysis seem adequate. The discussion comprises the essential topics. This manuscript provides the updated evidence to the readers. The study extends our knowledge of this correlation to an Asian country having a well-organized health care system where such an analysis could be performed based on a reliable database.

We conclude that oral corticosteroids use is associated with a trivial but statistically significant increase in the risk of hip fracture in the elderly in Taiwan. Additionally, the results suggest that there are dose-response and duration-response effects of oral corticosteroids on the risk of hip fracture. These findings confirm our understanding of oral corticosteroid-associated hip fracture in the elderly.

## Author Contributions

S-WL planned and conducted this study. He contributed to the conception of the article, initiated the draft of the article, and revised the article. C-LL conducted the data analysis and revised the article. K-FL planned and conducted this study. He participated in the data and revised the article.

## Conflict of Interest Statement

The authors declare that the research was conducted in the absence of any commercial or financial relationships that could be construed as a potential conflict of interest.
